# Visual Laterality of Calf–Mother Interactions in Wild Whales

**DOI:** 10.1371/journal.pone.0013787

**Published:** 2010-11-03

**Authors:** Karina Karenina, Andrey Giljov, Vladimir Baranov, Ludmila Osipova, Vera Krasnova, Yegor Malashichev

**Affiliations:** 1 Department of Vertebrate Zoology, Faculty of Biology and Soil Sciences, Saint-Petersburg State University, St. Petersburg, Russia; 2 Laboratory of Marine Mammals, P.P. Shirshov Institute of Oceanology, Moscow, Russia; 3 Department of Embryology, Faculty of Biology and Soil Sciences, Saint-Petersburg State University, St. Petersburg, Russia; University of Queensland, Australia

## Abstract

**Background:**

Behavioral laterality is known for a variety of vertebrate and invertebrate animals. Laterality in social interactions has been described for a wide range of species including humans. Although evidence and theoretical predictions indicate that in social species the degree of population level laterality is greater than in solitary ones, the origin of these unilateral biases is not fully understood. It is especially poorly studied in the wild animals. Little is known about the role, which laterality in social interactions plays in natural populations. A number of brain characteristics make cetaceans most suitable for investigation of lateralization in social contacts.

**Methodology/Principal Findings:**

Observations were made on wild beluga whales (*Delphinapterus leucas*) in the greatest breeding aggregation in the White Sea. Here we show that young calves (in 29 individually identified and in over a hundred of individually not recognized mother-calf pairs) swim and rest significantly longer on a mother's right side. Further observations along with the data from other cetaceans indicate that found laterality is a result of the calves' preference to observe their mothers with the left eye, i.e., to analyze the information on a socially significant object in the right brain hemisphere.

**Conclusions/Significance:**

Data from our and previous work on cetacean laterality suggest that basic brain lateralizations are expressed in the same way in cetaceans and other vertebrates. While the information on social partners and novel objects is analyzed in the right brain hemisphere, the control of feeding behavior is performed by the left brain hemisphere. Continuous unilateral visual contacts of calves to mothers with the left eye may influence social development of the young by activation of the contralateral (right) brain hemisphere, indicating a possible mechanism on how behavioral lateralization may influence species life and welfare. This hypothesis is supported by evidence from other vertebrates.

## Introduction

Distinct roles of the brain hemispheres in processing of information are now well known as the basis for asymmetric reactions to various stimuli positioned to the sides of an individual [1]–[3]. These asymmetric reactions are usually aligned to one side in most individuals in populations, representing lateralized biases for a number of animal behaviors. Such population level behavioral lateralizations are well documented for a wide range of vertebrates from fish to mammals (for reviews see [1], [4]), and even found in a number of invertebrates, showing a gradual evolution of lateralizations from flatworms to vertebrates (reviewed in [5]). At least for the latter a common pattern of brain and behavioral lateralization is now well recognized [1], [6]. Likely, from the earliest steps of vertebrate evolution two main alternative functions were divided between the hemispheres: the left brain predominantly controls the behavior in routine situations, while the right brain specializes in responding to unpredictable changes in the environment [7]. If focusing specifically to the functions of the right cerebral hemisphere, it is preferentially involved in the control of a number of ecologically significant situations, such as various inter- and intraspecific interactions. Lateralized reactions to a model alarming stimulus demonstrated in a wide range of species are striking examples of right hemisphere specialization in control of danger detection [8]–[12].

Social interactions are important for the survival and welfare of humans along with that of most other animal species. Accumulated evidence demonstrates that lateralization does exist in different aspects of social behavior too, such as agonistic interactions, gregarious behaviour, or individual recognition (reviewed in [13]). For example, in tetrapods, but not in fish, more intraspecific aggressive reactions are directed to the conspecifics on the left than on the right side of an individual [14]–[17]. In fish, however, opposite to other vertebrates, aggressiveness is usually directed to the right [18]. However, a number of teleost fish species [19], [20] and anuran tadpoles [21] prefer to observe their own mirror reflections with their left eye, what indicates the prevalent role of the right hemisphere in recognition and responding to conspecifics. Strikingly, tadpoles not only react to their mirror images asymmetrically, but this continuous observation of conspecifics influences positively their growth and development [22], [23].

In birds and mammals the same right hemispheric specialization is reflected in perception of even more complex social stimuli. Recognition of familiar vs. unfamiliar conspecifics in chicks [24], [25], or individual face discrimination in monkeys and sheep [26], [27] is realized mainly in the right brain hemisphere. A type of human behavior, where laterality in perception may play a role, is the left-directed visual attention due to a preference by most women to hold their infants in their arms so that the infant's face is in their left visual hemifield [28]. These data clearly demonstrate existence of population-level lateralization in various social behaviors in a range of vertebrate species, suggesting a biological significance of such a phenomenon. Interestingly, in social species of fish the overall level of lateralization in different tasks may be higher than in solitary ones [29], [30]. The analogous prediction for lateralization in insects also stands for social vs. non-social species [31], [32]. These facts make a basis for a recently prevailing hypothesis on the origin of population-level lateralization in vertebrates [7], [13], which implies its relation to the need to maintain coordination among asymmetrical individuals in social behaviours [33] (but see [34] for a differing hypothesis). Mathematical modelling indeed shows that during prey–predator or intraspecific (competitive and cooperative) interactions, population-level lateralization can in principle arise as an evolutionarily stable strategy [35], [36]. However, there have been very few behavioral observations of laterality in social contacts provided under natural conditions in any vertebrate species. Hence, a particular role of laterality in visually guided natural social behavior is not fully understood.

Visual laterality in social interactions is easier to assess in animals with laterally placed eyes. Cetaceans are especially suitable for this kind of research for three reasons: high level of sociality and interactions between individuals, stronger isolation of brain hemispheres due to relatively less developed corpus callosum, and transfer of all the visual information from an eye first to the contralateral brain hemisphere [37]–[40]. In beluga whale (*Delphinapterus leucas*), in which, as in all whales and humans, a strong bond between mother and young remains for some years [41], social contacts are of great significance for the calf's survival. Here, we show that during social interactions between the calf and the mother, calves of this whale species use their visual system asymmetrically. We further propose a mechanism by which the behavioral lateralization can influence the animal life and welfare.

## Results and Discussion

**Figure 1:**
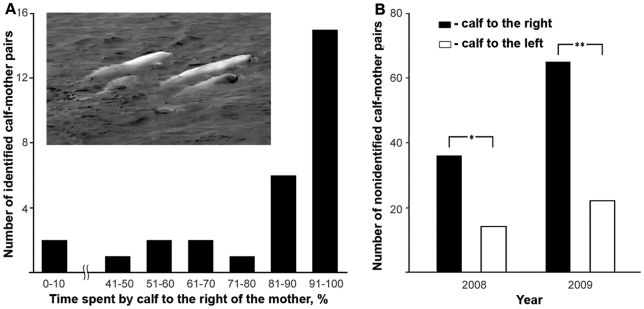
Position of calves in calf-mother pairs. (**A**) The distribution of 29 individually identified pairs depending on the percent of time spent by calf to the right of the mother (image insertion shows a view from the observation tower on two calves surfacing to the right of their mothers; see also Video S1). (**B**) Number of non-identified pairs registered during scans of the sea from the observation tower in two successive years (*G_1_ = 9.3, *P* = .0023; **G_1_ = 22.22, *P*<.0001).

**Figure 2:**
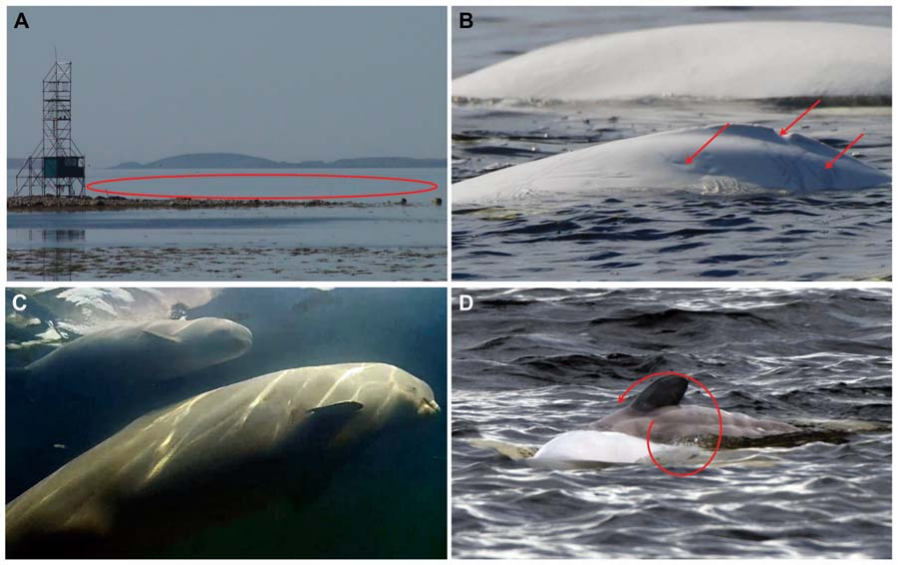
Aspects of the field study of lateralization in belugas. (A) Observation area in front of the observation tower is marked, but not restricted to the red elipse. (B) Characteristic markings on the body of a characteristic female used for individual identification marked with red arrows (from left to right: carvings, indent on the dorsal fin, scratches of different colour). (C) A young calf swimming with the mother in the view of underwater video camera. (D) A young calf rolling along the axis to the right of the mother. Red spiral arrow shows the direction of rolling.

We videotaped the social interactions of 29 individually identified wild beluga's calf–mother pairs. With one exception, the individual calves swam or rested significantly longer on a particular side of the mother (Chi-square tests 7.87 to 1140.09, *P*<.005), with significantly more calves showing a right-side than a left-side preference (26 out of 28; *G*1 = 24.41, *P* = .0001; Fig. 1A, Video S1). For the entire group, the mean percent of time swimming and/or resting also was significantly longer on the right of the mother during the whole period (81,4±4.87 (mean ± MSE); *t_28_* = 6.45, *P*<.0001) and the first minute of video recordings (86.32±5.17; *t_28_* = 7.03, *P*<.0001). The right-side calf-to-mother position also was preserved during mother and calf joint diving (non-identified pairs), as revealed by underwater video recordings (Fig. 2C) in 33 out of 43 episodes (77%).

The number of left- and right-positioned calves in individually non-identified pairs simultaneously present in the observation area has shown a highly significant right-side population bias in two successive years (Fig. 1B). Randomized observations from 80 non-identified pairs made in 2001 [41] also revealed a right-side population bias in the time spent by calf (%) relative to the mother (73.40±3.36 (mean ± MSE); *t_79_* = 6.96, *P*<.0001). This bias evidently represents the contributions of a very high percentage of individuals, all of them significantly lateralized in the same direction. To ensure that the observer position or direction of sea current do not affect calf's position, we analyzed randomly chosen 10-second video fragments for each pair of those recorded swimming both directions along the shore (N = 14), which would show the pair swimming leftward and rightward. The ratio between the total time the calves spent on the mothers left or right side during these leftward and rightward swims were analyzed using two*-*sample *F-*test for variance. Since no significant differences were found (*F_(1,27)_* = *1.18, P* = .*2870*) the data were further analyzed regardless of the particular direction of swimming of a pair.

For correct interpretation of the results, it is necessary to understand whether the calf or the mother is responsible for the positional asymmetry in a pair. Frame by frame analysis revealed that the positional asymmetry is definitely a result of the calf's, not the mother's, preference for observing the other with one eye. The beluga calf always takes the lead in choosing the position near the mother, e.g., after a rapid change of the direction of the pair's swimming. Observations of the pairs at rest revealed that the calf often continues to swim around the mother, while the latter stays motionless and probably sleep (Video S2). In such situations registered for 9 individually identified pairs in 2010, in all pairs the calf swam at the right side of the resting mother significantly longer time, than at the left side (Chi-square tests ranging from 5.24 to 124, *P*<.022). These observations testify in favor of the calf's prevailing role in choosing the position in relation to the mother. Although one can not exclude the possibility that the mother may monitor the calf with her right eye, it is nonetheless unlikely that the overall bias is due to the mother's tendency to keep the calf to one of her sides. Indeed, in dolphins these are calves that prefer certain positions to mother when frightened, threatened, or tired [42]. Unlike terrestrial vertebrates they demonstrate a higher degree of independence in deciding when and where to move [43] and perform most of the approaches and leaves in the calf-mother pairs [44]. Although dolphin mothers seem to be partially responsible for maintaining proximity to their calves, displaying more approaches than leaves in the pair [44], there is no evidence that mothers in either dolphins or belugas use just eye monitoring of the calves for that.

Furthermore, much like in belugas, dolphin mother-calf pairs maintain continuous visual contact with one another [45]. Remarkably, during monocular sleep, the eye that the dolphin calf directs toward the mother is open more often than is the other eye. This suggests that visual contact in calf-to-mother interactions is more important than tactile contact. That the beluga calf more often demonstrated activity (climbing on to the mother's back, rolling along the longitude axis, or touching the mother with the pectoral flipper while keeping the mother in its left visual hemifield (Fig. 2D; 61 of 74 cases, 82%, Chi-square test 31.135, P<0,0001), is further evidence of a calf visual preference. For flipper-to-body contacts this seems to be the case also in another species, the bottlenose dolphin, *Tursiops aduncus* [46]. A remarkable exclusion from this rule is found in sperm whale, *Physeter macrocephalus* [47]. Sperm whale calves peduncle diving is laterally asymmetrical with a bias to the left, and not to the right side of the escorting adult. This lateralization, however, may be a result of a unique nasal structure in this species (the blowhole is displaced to the left, while the right nostril is skinned over), and as a consequence, a probable nasal suckling [47], since such a leftward bias was only registered during calves' diving to reach the peduncle. In any case, this example also demonstrates the prevalent role of the calf in the choice of the side near to the mother.

From two weeks of age on beluga calves periodically leave their mothers and form transient associations with other individuals in the aggregation [41]. In dolphins such mother and calf separations was showed to play an important role in calf's socialization [43]. Obviously, during these contacts a calf displayed interest in and approached elders and thus chose the location as regards to the group. Importantly, when escorting long-lasting groups of much elder young whales young calves also exhibit the right-sided positional asymmetry of similar level as when escorting their mothers (24 out of 29 episodes, 83%, Chi-square test 12.448, P = 0.0004). The fact that beluga calves prefer to keep at the right of elder calves and possibly observe them with the left eye, indicates that the laterality effect occurs not exclusively in response to the mother but may extend to other socially significant objects. A further plausible suggestion is that elder calves or may be even adults of this and other species may also prefer to approach one another from right to form a group or to join a preexisting group. This hypothesis can be checked in future in whales possessing well recognized natural individual marking (belugas, killer whales).

Recently, a number of reports has shown the right eye/left hemisphere advantage for certain visually guided tasks in dolphins, particularly in a test for numerical abilities and in a multiple pattern discrimination task [48]–[50]. In contrast, our data, showing a left eye preference during calf-mother interactions in belugas, together with an earlier report on left-sided bias in dolphin's flipper-to-body contacts [46] indicate that the analysis of socially significant visual information occurs in whales in the right brain hemisphere. This is in accordance with what is known for other vertebrates, e.g., chick [51]–[52], or fish [53]. As we have shown recently, left eye – right hemisphere system is also involved in discrimination of novel objects in beluga whales [54], again demonstrating a similarity to other vertebrates [55]–[57]. In addition, several studies of foraging dolphins [58] and whales [59] under natural conditions revealed a number of right-sided preferences suggesting a complementary role of the left hemisphere for feeding behavior. Previously the same bias was found in a number of both land [60]–[62], and aquatic vertebrates [31], [63], [64]. Hence, processing of information on social partners or novel objects vs. food correspondingly in the right and the left brain hemispheres in whales is, therefore, in line with the stimulus–specific pattern of brain lateralization common to all vertebrates [1], [4], [13], [65]. Hence, these basic left/right hemisphere specializations are expressed in the same way in cetaceans and other vertebrates. The existing disagreement [48]–[50] might be a matter of different possible interpretations or experimental design and needs further investigation.

The occurrence of striking population–level lateralization in such a highly social species as beluga whale is consistent with a mathematical model, predicting that animals with prevalence of synergistic over antagonistic interactions should display most strong population bias [36]. However, the exact mechanism, which governs the alignment of the behavioral asymmetry in population, is not known. We believe that continual unilateral eye contacts of beluga whale calves first to their mothers and later to other conspecifics may promote the development of cognitive-communicational skills via preferential activation of the right hemisphere. Hence, the calves with the left eye/right hemisphere preference receive more chances for better performance and survival. The same could be true for primate infants (and actually, not necessary restricted to them) who, being held by their mothers preferentially on the left, spend more time looking at the mother's face with the left eye than with the right [66]–[68]. A number of hypotheses have been put forward in order to explain left-sided bias in cradling the infants in humans and its possible influence on development of handedness in children [66], [67]. However, it is still unclear why such a bias exists and what might be its benefits for the mother or for the infant. Although more often and straightforward explanations relate it to the handedness of the mother, which may influence in this or that way the handedness of the infant, it is more credible that multiple causes may act here. Among others a role might have the emotional state of the mother attending the socially significant object (the infant) [28], [67], a preference of the child to listen the mothers heart beat [69]–[71] or even to observe the mother's face with the left eye [72]. Indeed, as hypothesized by the latter authors, left-side cradling may probably facilitate perceptual communication between mother's and infant's right cerebral hemispheres [72]. More important is that regardless of its particular reason, the side of cradling may indeed influence the overall development of the young by means of its already established brain asymmetry, i.e., by activating one of the differently specialized hemispheres (the right one). Interestingly, a real phenomenon of right hemisphere activation with unilateral eye stimulation in experiments with non-primate mammals was explicitly shown by others [73], [74]. For example, cows, which chronically receive food from the left, which thus appear first in their left visual hemifield and is analyzed in the right hemisphere improve their lactation and breeding performance. Similarly, tadpoles of frogs, which prefer to observe their mirror images and conspecifics with the left eye, grow faster and develop better in mirrored aquaria than in those with the opaque walls [22], [23]. All these facts together indicate an important mechanism by which the left eye/right brain hemisphere system may influence the species' life and welfare.

## Materials and Methods

### 1. Region and season of field work; observation conditions

Observations on whales were conducted at one of the greatest belugas' breeding aggregation at the Beluzhiy Cape (35.52N 65.07E) of the Solovetskiy Island (Onega Bay, Southern part of the White Sea). The observations on belugas here have been performed since 1995, so that the whales are aware of presence of humans and demonstrate natural behavior. This aggregation is formed mostly by females with calves of various ages [75]. The aggregation is uniquely close to the shore (12–25 m) so that the observations are possible either directly from the shore line, or from the observation tower (12 m height; Fig. 2A). The observations were carried out every time belugas came to the studied area, i.e., once or twice a day at low-tide [76], except adverse weather conditions. Data on individually identified mother–calf pairs and underwater recordings of unidentified animals were collected in July–August 2009; population scans (see below) have been repeatedly made in 2008 and 2009. Data on individually non-recognized pairs were also collected in June-August 2001. Additional observations on calves' behavior, when escorting mothers and elder calves were made in July–August 2010.

#### Ethics statement

This study does not include any study of human subjects or non-human primates, thus does not need any specific adherence to the Declaration of Helsinki or Weatherall report. As for the work with other subjects, this work, which only implies pure observations on animals, did not require any permission according to local rules and laws in Russia.

### 2. Individually identified mother–calf pairs

The individual identification of adult belugas and mother-calf pairs was carried out using natural markers (coloration pattern, scars, and fin injuries, Fig. 2B). 17 pairs were observed once (during one day) each in the studied area, 8 pairs – twice, two pairs – three times, and two pairs – four times. We continually video recorded mother–calf pairs while they were joint swimming (within 4 m one from the other) or resting in the observation area directly from the shore as long as possible. For each pair the time spent by calf to the left or to the right of the mother was scored. Near to one half of all the individually identified pairs were observed more than one time (one low-tide). Heterogeneity chi-square tests were performed to allow pooling data from different days (low-tides). The first minute of video from each pair was included into analysis; the data from pairs recorded for less than 1 minute were discarded. Mean population time spent on one or the other sides of the mother were compared using paired Student's t-test (N = 29). Analysis at the individual level was performed throughout all recording time using Chi-square tests. Thereafter the number of calves displaying individual preference to swim to the left side of the mother was compared with the number of calves significantly preferred to swim to the right side using the log-likelihood ratio chi-square test (G-test). Tactile contacts initiated by the calf in each calf-mother pair were scored separately.

### 3. Individually non-identified calf-mother pairs

To register the position of the calf in individually non-recognized pairs we scanned the observation area from the tower. The scanning was performed three times per low-tide period at approximately 30–40 minute intervals. All visible mother–calf pairs swimming in the observation area were registered and the calf's position was scored.

To check whether position of the calf preserves when diving, a digital camera in waterproof box was installed 15 m off the shore, at the depth 5 m and directed towards the main area, where whales usually swam. Located on the observation tower camcorder recorded the video receiving from underwater camera. Left or right calf-to-mother position was scored every time a pair got into the camera capture field (totally 43 episodes). Individual identification was impossible due to light insufficiency. The total number of left and right sided calf-to-mother registrations was scored, and the population bias was estimated using G-test.

## Supporting Information

Video S1 Mother-calf joint surface swimming. This video illustrates a typical episode of a calf swimming to the right of the mother, along with other activities of the calf, i.e., tactile contacts described in the main text.
(10.04 MB AVI)

Video S2 Mother-at-rest and calf surface interactions. This video illustrates a typical behavior of a calf swimming along the resting and mostly motionless mother.
(10.17 MB AVI)
